# Psychosocial Functioning, Dietary Patterns and Socioeconomic Determinants of Quality of Life in Polish Women with Fibromyalgia: A Cross-Sectional Study

**DOI:** 10.3390/nu18132081

**Published:** 2026-06-25

**Authors:** Robert Gajda, Marzena Jeżewska-Zychowicz

**Affiliations:** 1 Department of Human Nutrition, Faculty of Biotechnology and Food Sciences, Wrocław University of Environmental and Life Sciences, Chełmońskiego 37, 51-630 Wroclaw, Poland; 2 Department of Food Market and Consumer Research, Institute of Human Nutrition Sciences, Warsaw University of Life Sciences (SGGW-WULS), Nowoursynowska 159C, 02-776 Warsaw, Poland; marzena_jezewska_zychowicz@sggw.edu.pl

**Keywords:** fibromyalgia, quality of life, psychosocial functioning, dietary patterns, socioeconomic determinants, women

## Abstract

Background: Fibromyalgia is a chronic pain syndrome associated with impaired psychosocial functioning, reduced quality of life, and substantial socioeconomic burden. Although nutritional approaches to fibromyalgia management are increasingly investigated, the relationships between dietary patterns, socioeconomic determinants, and quality of life remain unclear. This study aimed to assess associations between fibromyalgia severity, psychosocial functioning, dietary patterns (DPs), socioeconomic factors, and self-rated quality of life among Polish women with fibromyalgia. Methods: A cross-sectional online survey was conducted between March and April 2026 among members of nationwide Polish Facebook support groups for individuals with fibromyalgia. The final analysis included 201 women who self-reported a physician diagnosis of fibromyalgia. Fibromyalgia severity was assessed using the Modified Fibromyalgia Assessment Status (FAS), disease impact using the Revised Fibromyalgia Impact Questionnaire (FIQR), and quality of life using selected WHOQOL-BREF domains. Dietary patterns were identified using principal component analysis (PCA) based on the frequency of consumption of 33 food groups from the KomPAN questionnaire. Hierarchical linear regression models were applied to identify predictors of self-rated quality of life. Results: Four dietary patterns were identified: traditional, meat-and-fat, pro-healthy, and dairy-based. Participants reported low overall quality of life and moderate fibromyalgia severity. A better self-rated financial situation was associated with a higher quality of life only in the initial regression model (β = 0.325; *p* < 0.001). After adjustment for clinical and psychosocial variables, socioeconomic factors and dietary patterns were no longer significant predictors. Functional limitations (FIQR Functioning: β = −0.202; *p* = 0.022) and overall disease impact (FIQR Overall Impact: β = −0.189; *p* = 0.026) were negatively associated with quality of life, whereas psychological (β = 0.234; *p* < 0.001) and social functioning (β = 0.162; *p* = 0.023) were positive predictors. The final model explained 40.3% of the variance in quality of life. Conclusions: These findings support a comprehensive biopsychosocial model of fibromyalgia care.

## 1. Introduction

Fibromyalgia is a chronic pain syndrome characterised by widespread musculoskeletal pain accompanied by symptoms such as fatigue, sleep disturbances, and cognitive dysfunction. Its pathogenesis is believed to be associated with abnormalities in central pain processing within the central nervous system and increased pain sensitivity [1].

The global prevalence of fibromyalgia is currently estimated at approximately 2–8% of the general population, although these figures vary depending on the diagnostic criteria applied and the population studied [2]. The condition occurs considerably more frequently in women, with a female-to-male ratio estimated at 7–9:1. It is most commonly diagnosed in middle adulthood, although it may occur at any stage of life [2]. Recent data from Poland indicate that the prevalence of fibromyalgia is approximately 2–4%; however, these estimates may be underestimated due to diagnostic challenges and limited accessibility to specialist assessment [3].

Fibromyalgia significantly reduces patients’ quality of life, negatively affecting physical, psychological, and social functioning [4]. The principal factors contributing to impaired quality of life include pain severity, fatigue, and sleep disturbances, all of which directly limit daily activity and functional capacity [5]. In addition, psychological factors such as depression, anxiety, and maladaptive coping strategies substantially exacerbate quality of life impairment and mediate the impact of somatic symptoms [4]. Evidence suggests that appropriate therapeutic interventions may lead to significant improvements in quality of life through symptom reduction and enhanced psychosocial functioning [6].

Individuals with fibromyalgia are more likely to present with a lower socio-economic status, including lower levels of education and income, which may adversely affect access to healthcare and treatment effectiveness [7]. The condition also substantially limits work capacity, leading to increased absenteeism, reduced productivity, and a higher risk of unemployment and work disability [8]. Both the direct and indirect costs associated with fibromyalgia, including healthcare expenditure and loss of income, constitute a considerable economic burden for patients and healthcare systems alike [9]. Furthermore, social factors such as social support and living conditions play a crucial role in modulating disease progression and the quality of life of individuals with fibromyalgia [7].

Dietary patterns among individuals with fibromyalgia frequently deviate from nutritional recommendations and are characterised by irregular meal structures and the consumption of foods with pro-inflammatory potential [10]. At the same time, some patients tend to modify their diet following diagnosis, including restricting selected food groups such as cereals, dairy products, or fish, which may increase the risk of nutritional deficiencies [11]. Evidence suggests that anti-inflammatory dietary patterns, including the Mediterranean, plant-based, and antioxidant-rich diets, may be associated with reduced pain severity, fatigue, oxidative stress, and overall symptom burden in individuals with fibromyalgia [10,11,12,13,14,15,16]. Other nutritional strategies, such as low-FODMAP diets, micronutrient supplementation, and modulation of the gut microbiota, are increasingly being investigated as components of a comprehensive therapeutic approach to fibromyalgia management, although the quality of evidence remains heterogeneous and further randomized controlled studies are warranted [17,18,19,20,21].

Despite the growing body of research on fibromyalgia, there remains a lack of comprehensive analyses simultaneously integrating clinical, nutritional, and socio-economic factors, which limits a full understanding of the multifactorial nature of the condition [22]. Current studies investigating the role of diet in fibromyalgia suggest potentially beneficial associations; however, findings remain inconsistent, and the quality of evidence is limited, highlighting the need for longitudinal and multicentre research [23,24,25]. Importantly, improvements in clinical symptoms do not necessarily translate into better overall quality of life, which represents a multidimensional construct encompassing physical, psychological, social, and environmental domains. Consequently, quality of life may be influenced more strongly by psychosocial functioning and disease-related functional limitations than by dietary habits alone [4,5]. Furthermore, cross-sectional studies have not yet sufficiently explored population-specific differences, including national contexts such as Poland, nor the interrelationships between socio-economic determinants, quality of life, dietary patterns, and the severity of fibromyalgia symptoms [22].

In this context, the following hypotheses were proposed: (1) self-rated overall quality of life is lower in older individuals, individuals with poorer economic status and higher BMI; (2) dietary patterns are not associated with self-rated overall quality of life among patients with fibromyalgia; (3) self-rated overall quality of life is lower in individuals with greater fibromyalgia severity; (4) self-rated overall quality of life is higher in individuals with better psychological and social functioning. Therefore, the aim of the study was to assess the relationships between fibromyalgia symptom severity, psychosocial functioning, dietary patterns, socio-economic determinants, and self-rated quality of life among adult women with fibromyalgia in Poland.

## 2. Materials and Methods

### 2.1. Study Design and Sample

A cross-sectional study was conducted in March and April 2026 among individuals with fibromyalgia who were members of nationwide Facebook support groups, including “Fibromialgia”, “Razem w Fibromialgii”, and the “Fibro-My” Association. Participants active in these groups were invited to complete an online questionnaire created using Google Forms. A total of 236 questionnaires were collected. Of these, 35 responses were excluded due to lack of informed consent to participate in the study (n = 2), declaration of not having fibromyalgia (n = 3), absence of self-reported physician diagnosis of fibromyalgia (n = 10), incomplete questionnaire data (n = 13), and male sex (n = 7), owing to the low representation of men in the study sample. Consequently, 201 completed questionnaires from women were included in the final analyses.

The study was conducted in accordance with the Declaration of Helsinki. Participation in the survey was voluntary. Informed consent was obtained from all participants. The study was approved by the Rector’s Committee for Research Ethics of the Wrocław University of Environmental and Life Sciences on 16 March 2026, following an opinion on the research project’s compliance with ethical principles (Resolution No. N0N00000.0020.1.3.5.2026).

### 2.2. Questionnaire

The occurrence and severity of fibromyalgia were assessed using the Modified Fibromyalgia Assessment Scale (FAS 2019 modCr) [26]. According to the interpretation adopted for this scale, the total score ranged from 0 to 39 points, including fatigue severity and sleep quality (0–20 points) and pain distribution (0–19 points). Higher scores indicated greater severity of fibromyalgia symptoms [26]. Although no separate Polish adaptation of the modified Fibromyalgia Assessment Scale has been published, the instrument has demonstrated good validity, reliability, and diagnostic agreement with the ACR criteria in European populations and was therefore considered appropriate for evaluating fibromyalgia severity in this study [26].

The functional status of individuals with fibromyalgia was assessed using the Revised Fibromyalgia Impact Questionnaire (FIQR) [27], based on the validated Polish version of the instrument (FIQ-Pol) [28]. The total FIQR score ranges from 0 to 100 points, with higher scores indicating a greater impact of the disease on the patient’s life [29,30,31]. The total score is calculated from three domains: in the “function” domain, the sum of points is divided by 3 (maximum 30 points); in the “overall impact” domain, points are summed directly (maximum 20 points); and in the “symptoms” domain, the sum of points is divided by 3 (maximum 50 points).

Self-rated quality of life was assessed using the following question from the WHOQOL-BREF (World Health Organization Quality of Life—Brief) questionnaire [32]: “How would you rate your quality of life?”, with the following response options: 1—very poor; 2—poor; 3—neither poor nor good; 4—good; and 5—very good. In addition, psychological functioning (WHOQOL-BREF psychological domain) and social functioning (WHOQOL-BREF social domain) were assessed using the same questionnaire. Higher scores indicated better subjective quality of life within the respective domain [32]. The WHOQOL-BREF was administered using the officially validated Polish version, for which psychometric evaluation conducted in a representative sample of 908 Polish respondents confirmed satisfactory construct validity and good internal consistency across all domains (Cronbach’s α ranging from 0.69 to 0.81), supporting its use in health-related quality of life research [33].

Dietary patterns were identified based on the frequency of consumption of 33 food and beverage groups [34]. Respondents reported their habitual intake frequency for each food item over the 12 months preceding the study using one of the following response categories: 1—less than once a month or never; 2—1–3 times per month; 3—once a week; 4—several times a week; 5—once a day; or 6—several times a day.

Questions regarding age, height, body weight, and self-rated economic status (“How would you assess your financial situation?”, with the following response options: 1—I live in poverty; 2—I live very modestly; 3—I live moderately; 4—I live well; 5—I live very well were derived from the KomPAN questionnaire [34]. Data on height and body weight were used to calculate body mass index (BMI) [35].

### 2.3. Statistical Analysis

Descriptive statistics were used to present the demographic characteristics of the study sample and the variables included in the analyses. Factor analysis was performed to identify dietary patterns (factors) based on the frequency of consumption of 33 food and beverage groups. Following orthogonal rotation (Varimax rotation with Kaiser normalisation), the number of factors was determined using the following criteria: eigenvalues > 1.0, scree plot analysis, and factor interpretability. Food products were considered to load on a factor if they demonstrated an absolute correlation coefficient of 0.4 or higher. Factors were extracted using principal component analysis (PCA), with variables describing daily food consumption frequency entered into the model. The suitability of the data for factor analysis was confirmed by a Kaiser–Meyer–Olkin (KMO) measure of sampling adequacy of 0.671 and Bartlett’s test of sphericity (*p* < 0.001). The names assigned to the identified dietary patterns were based on the food groups with the highest factor loadings, in accordance with the widely accepted principal component analysis methodology [36,37]. The labels “Traditional”, “Meat-and-fat”, “Healthy”, and “Dairy” reflect the predominant food groups characterizing each pattern and are consistent with the terminology used in previous dietary pattern studies conducted in European populations, including studies employing the KomPAN questionnaire [34,36,37]. Four dietary patterns (factors) were identified, each explaining more than 5% of the variance: factor 1—traditional pattern; factor 2—meat-and-fat pattern; factor 3—healthy pattern; and factor 4—dairy pattern (Table 1). Food items that did not correlate with any of the identified factors and were therefore not included in Table 1 comprised: dishes prepared from legumes, e.g., beans, peas, soybeans, and lentils; canned meat products; fruit juices; vegetable juices; dishes prepared from so-called white meat, such as chicken, turkey, or rabbit; fish-based dishes and snacks; energy drinks (e.g., KC, Black Horse, Red Bull, Burn, Shot, or similar products); carbonated and non-carbonated soft drinks such as Coca-Cola, Pepsi, Sprite, Fanta, orangeade, or lemonade; lard or bacon fat used as a spread or for frying and baking; egg-based dishes and snacks; canned fruit and vegetables, pickled or fermented vegetables; alcoholic beverages such as beer, wine, and vodka; powdered soups or ready-made soups (e.g., canned, jarred, or condensed, excluding frozen soups); and fast food products (e.g., hot dogs, baguettes, pizza, chips, hamburgers, etc.).

Hierarchical linear regression analysis was performed to assess the associations between perceived overall quality of life and socio-economic variables, dietary patterns, indicators describing the functioning of individuals with fibromyalgia, and their social and psychological functioning. Independent variables were entered into the regression equation in a purposeful sequence, beginning with variables characterising the study group (age, BMI, and self-rated economic status) (Model 1), followed by dietary patterns (Model 2), variables describing fibromyalgia (Model 3), and finally variables related to social and psychological functioning (Model 4). A *p*-value of <0.05 was considered statistically significant.

A post hoc power analysis was performed for the final hierarchical regression model according to Cohen’s methodology [38]. Assuming a significance level of α = 0.05, a sample size of 201 participants, 13 predictors, and an observed coefficient of determination (R^2^ = 0.442), the statistical power exceeded 0.99, indicating that the study was adequately powered to detect the observed associations. According to Cohen’s criteria, an effect size of f^2^ = 0.79 (calculated as R^2^/(1 − R^2^)) represents a large effect [38,39].

All analyses were performed using IBM SPSS Statistics, version 31.0 (IBM Corp., Armonk, NY, USA).

## 3. Results

The characteristics of the study group are presented in Table 2. The study sample comprised 201 individuals, with the largest proportions aged 46–55 years (33.3%) and 36–45 years (29.4%). The majority of respondents had higher education (60.7%) and lived in cities with more than 100,000 inhabitants (47.8%). Most participants were in a marital or partner relationship and shared a household either with a partner only (39.8%) or with both a partner and family members (34.8%). Nearly half of the respondents reported a moderate financial situation (48.2%), while 29.9% reported a good financial situation. Half of the participants were in permanent employment (50.2%). More than one-third of respondents (34.9%) reported receiving financial support from family members due to economic difficulties. Most participants (58.7%) reported not requiring social welfare support despite financial difficulties, and 65.2% did not receive such support despite reporting health-related problems (Table 2).

Data presented in Table 3 indicate that individuals with fibromyalgia demonstrated a low self-rated overall quality of life (mean = 2.42 ± 0.85 points). At the same time, the scores obtained in the psychological (10.52 ± 1.86 points) and social (10.61 points) domains of the WHOQOL-BREF suggest poor quality of life across both emotional functioning and social relationships.

The mean FAS score indicating fibromyalgia severity was 25.02, corresponding to a moderate level of disease severity according to the study’s classification. Simultaneously, the high FIQR scores, particularly in the symptoms domain (35.37 points) and functioning domain (20.08 points), indicate substantial clinical symptom burden and a marked impact of fibromyalgia on respondents’ daily functioning (Table 3). Analysis of dispersion measures demonstrated relatively high variability in the results, especially in the social domain of quality of life and in the severity of fibromyalgia symptoms. Furthermore, the skewness values suggest an asymmetric distribution for some variables, particularly the quality-of-life variable in the social domain and the FAS score (Table 3).

Table 4 presents the results of the hierarchical linear regression analysis examining the associations between self-rated overall quality of life and selected socio-economic variables, dietary patterns, fibromyalgia severity, and psychosocial functioning among individuals with fibromyalgia. The analysis demonstrated that the explanatory power of the models increased progressively with each step, from 9.9% in Model 1 to 40.3% in the final model, indicating the substantial importance of clinical and psychosocial factors in shaping respondents’ perceptions of quality of life.

In Model 1, which included the variables from the first hypothesis, the only significant predictor of self-rated quality of life was self-rated economic status (β = 0.325; *p* < 0.001), indicating that a better financial situation was associated with higher perceived quality of life. Neither age nor BMI showed significant associations with the dependent variable analysed.

After the inclusion of dietary patterns in Model 2 (variables included in the second hypothesis), no significant associations were observed between any of the identified dietary patterns and self-rated quality of life. At the same time, the significant positive association between quality of life and self-rated economic status remained unchanged (β = 0.311; *p* < 0.001).

In Model 3, following the inclusion of fibromyalgia-related variables (the third hypothesis), namely FIQR Functioning, FIQR Overall Impact, FIQR Symptoms, and FAS, the FIQR domains related to functioning (β = −0.202; *p* = 0.030) and the overall impact of the disease on patients’ lives (β = −0.220; *p* = 0.015) emerged as statistically significant. This indicates that greater functional limitations and a stronger impact of the disease on daily life were associated with lower self-rated quality of life. In contrast, the FAS score and the FIQR symptoms domain did not reach statistical significance. Moreover, after adjustment for clinical variables, economic status was no longer a significant predictor of quality of life.

Model 4, supplemented with the social and psychological domains of quality of life (variables included in the fourth hypothesis), demonstrated the highest proportion of explained variance (adjusted R^2^ = 0.403). In this model, the psychological (β = 0.234; *p* < 0.001) and social (β = 0.162; *p* = 0.023) domains of quality of life emerged as significant positive predictors of self-rated overall quality of life. This indicates that better psychological and social functioning was associated with a higher overall quality of life among the participants. At the same time, a significant negative effect of fibromyalgia-related functional limitations (FIQR Functioning; β = −0.202; *p* = 0.022) and the overall impact of the disease (FIQR Overall Impact; β = −0.189; *p* = 0.026) remained evident.

In the final model, no significant effects of age, BMI, dietary patterns, or fibromyalgia severity (FAS score) on self-rated quality of life were observed.

Table 5 presents a summary of the successive hierarchical regression models explaining self-rated overall quality of life among individuals with fibromyalgia. Model 1, including age, BMI, and self-rated economic status, explained 11.3% of the variance in quality of life (R^2^ = 0.113; adjusted R^2^ = 0.099). This indicates that basic socio-economic characteristics made a limited, though statistically significant, contribution to participants’ perceptions of quality of life. The inclusion of dietary patterns in Model 2 resulted in only a slight increase in the coefficient of determination (R^2^ = 0.124), with an increment in explained variance of merely 1.2% (ΔR^2^ = 0.012). At the same time, the adjusted R^2^ decreased slightly (0.092), suggesting that dietary patterns did not meaningfully explain self-rated quality of life among individuals with fibromyalgia.

The greatest increase in explained variance was observed in Model 3 following the inclusion of fibromyalgia-related variables (FAS and FIQR domains). The R^2^ value increased to 0.354, while the increment in explained variance reached 23.0% (ΔR^2^ = 0.230). These findings indicate that clinical factors related to functioning and the impact of the disease constitute key determinants of quality of life among patients with fibromyalgia.

Model 4, extended to include scores describing quality of life in the social and psychological domains, achieved the highest level of explained variance (R^2^ = 0.442; adjusted R^2^ = 0.403). The addition of psychosocial variables increased the explained variance in quality of life by a further 8.8% (ΔR^2^ = 0.088). These findings indicate that psychological and social functioning play a significant role in the perception of overall quality of life among individuals with fibromyalgia and provide a stronger explanation of quality of life than dietary patterns, age, self-rated economic status, or BMI (Table 5).

## 4. Discussion

The aim of the study was to assess the relationships between fibromyalgia symptom severity, psychosocial functioning, dietary patterns, socio-economic determinants, and self-rated quality of life among adult women with fibromyalgia in Poland. The findings demonstrated that perceived quality of life was primarily associated with self-rated economic status, the impact of fibromyalgia on daily functioning (FIQR Functioning, FIQR Overall Impact), and psychological and social functioning. Hierarchical regression analysis further revealed that psychosocial and functional factors accounted for the greatest proportion of variance in overall quality of life, thereby supporting the biopsychosocial nature of fibromyalgia [1,5].

The study group demonstrated a moderate severity of fibromyalgia, consistent with previous epidemiological and clinical studies reporting that patients with fibromyalgia typically present with moderate symptom burden and functional impairment in community and clinical populations [26,40,41]. Low scores were also observed in the psychological and social functioning domains, which may be attributed to the chronic nature of the condition leading to social isolation, deterioration of interpersonal relationships, and reduced occupational and social activity [7,8]. These findings emphasise the need for comprehensive interventions addressing not only physical symptoms but also psychological and social support, consistent with the biopsychosocial model of fibromyalgia, which recognises the interaction between biological mechanisms, psychological processes, and social determinants in shaping symptom severity, functional impairment, and quality of life [6,42,43].

Functional limitations associated with fibromyalgia have been shown to be one of the factors strongly related to poorer quality of life [7]. Their significance remained evident even after accounting for quality of life in the psychological and social domains. Our research indicates that impaired physical functioning and a greater impact of the disease on daily activities are more strongly associated with psychological well-being than pain severity alone, suggesting that functional limitations constitute a key determinant of mental health and quality of life in individuals with fibromyalgia [5,42,44,45]. This may explain the lack of association observed between age, BMI, and perceived quality of life [4].

Despite previous reports indicating that greater symptom severity is associated with poorer quality of life [28,46], the present study did not demonstrate an association between quality of life and either the FAS score or the FIQR symptoms domain. These findings suggest that functional limitations may play a more important role in assessing quality of life than somatic symptoms alone [4]. The significance of self-rated economic status diminished after adjustment for clinical variables, indicating that this relationship may be partially mediated by functional impairment and the impact of the disease on daily life [8]. The obtained results are consistent with the biopsychosocial model of fibromyalgia [1].

No significant associations were identified between dietary patterns and self-rated quality of life in this cross-sectional study. It cannot be interpreted as evidence that healthy eating is unimportant for women with fibromyalgia. Rather, our findings suggest that, within the biopsychosocial framework of the disease, psychosocial well-being and functional capacity are more strongly correlated with perceived quality of life than habitual dietary patterns. Therefore, maintaining a generally healthy lifestyle, including a balanced diet, should be regarded as an important component of overall health promotion, while interventions targeting psychological support and functional improvement may have a greater impact on quality of life [4,6,13].

The literature suggests that the beneficial effects of anti-inflammatory dietary interventions are primarily reflected in reductions in pain, fatigue, and overall symptom burden, whereas evidence for direct improvements in overall quality of life remains limited and inconsistent across studies [10,13,23,24]. Furthermore, the effectiveness of nutritional interventions remains inconclusive due to the heterogeneity of study populations and the coexistence of psychological and socio-economic factors [11].

The absence of associations between dietary patterns and quality of life may also be related to methodological considerations. Self-reported dietary assessment is susceptible to recall and reporting bias, potentially attenuating true associations [47]. Moreover, dietary patterns derived using principal component analysis are sample-dependent and may lead to some degree of participant misclassification, reducing the ability to detect modest dietary effects [36,37]. Residual confounding and limited statistical power to identify weak associations should also be considered as alternative explanations [48].

Psychological and social functioning demonstrated a positive association with perceived quality of life, consistent with previous studies on the well-being of individuals with chronic diseases [4]. At the same time, the relationship between functional limitations, the impact of the disease on daily life, and quality of life remained significant, whereas no association was observed with the severity of somatic symptoms [7]. These findings suggest that the psychosocial consequences of fibromyalgia may play a greater role in shaping patients’ well-being than clinical characteristics and dietary habits alone [1].

The obtained results partially confirmed the first research hypothesis, as poorer economic status was associated with lower quality of life only in the initial regression models [7,8]. The second hypothesis was likewise confirmed, since dietary patterns were not associated with quality of life [10,11]. The association between the impact of fibromyalgia on functioning and perceived quality of life was also confirmed, in line with the biopsychosocial model of the disease [1,5]. The fourth hypothesis was clearly supported, as better psychological and social functioning emerged as significant predictors of quality of life [4,6]. Overall, the findings indicate that the quality of life of women with fibromyalgia is primarily linked to the psychosocial and functional consequences of the disease [1,4].

In light of the obtained findings, the implementation of multidisciplinary therapeutic programmes incorporating psychological support, health education, and interventions aimed at improving the social functioning of women with fibromyalgia appears to be justified [1,6]. Reducing economic barriers to treatment and rehabilitation may also be of considerable importance [4,7]. Furthermore, therapeutic strategies should focus on improving functional capacity and on promoting adaptive coping mechanisms to manage chronic illness [5,6].

Although no direct association between dietary patterns and quality of life was identified in the present study, the literature suggests that anti-inflammatory diets may contribute to symptom reduction and improved functioning [10,12]. Increasing evidence indicates that diets with a low pro-inflammatory potential, particularly those based on the Mediterranean dietary model, may significantly reduce pain severity, fatigue, anxiety, and functional limitations in women with fibromyalgia through modulation of inflammatory processes and oxidative stress [10,12]. In a randomised controlled trial, Casini et al. demonstrated that a personalised Mediterranean diet improved quality of life and reduced pain-related disability, fatigue, and psychological symptoms in patients with fibromyalgia [10]. Similarly, Ersoy Söke et al. [12] confirmed that a higher pro-inflammatory dietary potential was associated with greater pain severity and a more severe disease course, suggesting that anti-inflammatory nutritional strategies may represent an important component of supportive therapy in fibromyalgia.

The principal limitation of the present study is its cross-sectional design, which allows the identification of associations but does not permit the determination of their directionality. Consequently, it cannot be conclusively established whether better psychosocial functioning and fewer functional limitations lead to a higher quality of life, or whether a higher quality of life contributes to improved psychosocial functioning and functional status. Therefore, future longitudinal and intervention studies are warranted to clarify the temporal sequence and causal nature of these relationships, thereby providing a stronger evidence base for developing targeted management strategies for women with fibromyalgia [49,50,51].

Although previous studies have shown that fibromyalgia is more common among individuals with lower socioeconomic status and lower educational attainment [7], the high proportion of participants with higher education (60.7%) in the present study is likely attributable to the online recruitment strategy through nationwide support groups. Internet-based recruitment preferentially attracts individuals who are more engaged in health information seeking, disease self-management, and peer-support activities, and who generally have higher educational attainment and health literacy, resulting in self-selection bias [52,53].

Another limitation relates to the use of self-reported data concerning diet, body weight, quality of life, and symptom severity, all of which may be affected by recall and response bias [11]. Moreover, fibromyalgia diagnosis was based on participants’ self-report of a previous physician diagnosis and was not independently verified by the investigators. Consequently, disease misclassification cannot be completely excluded, although self-reported physician diagnoses are commonly used in large epidemiological and online survey studies. Moreover, overall quality of life was assessed using a single global item from the WHOQOL-BREF rather than the full multidimensional construct. Although single-item measures demonstrate acceptable validity for assessing overall quality of life, they may be less sensitive to differences across physical, psychological, social, and environmental domains and generally show lower reliability than multi-item scales [54,55]. Therefore, the observed associations should be interpreted with appropriate caution.

In addition, the participants were recruited through online fibromyalgia support groups, which enabled access to women from different regions of Poland and facilitated the enrolment of individuals with a relatively low-prevalence chronic condition that is difficult to recruit through a single clinical centre [41,56]. Although this strategy increased the geographical diversity and heterogeneity of the study sample, it may have preferentially included women who are more actively engaged in disease self-management, health information seeking, and peer support, potentially introducing volunteer and self-selection bias [40]. Consequently, the findings should be interpreted primarily as applicable to an actively engaged population of women with fibromyalgia rather than to all individuals with the condition. However, because the primary objective of this cross-sectional study was to investigate associations between dietary patterns, psychosocial functioning, and quality of life rather than to estimate population parameters, the applied recruitment strategy is unlikely to substantially affect the internal validity of the observed relationships, although it may limit their external validity and generalisability [57,58]. Therefore, future multicentre and population-based studies are warranted to confirm the present findings. Moreover, the predominance of women with higher education in the study sample may further limit the generalisability of the findings to the broader population of Polish women with fibromyalgia, particularly those with lower socioeconomic status. Nevertheless, because the primary objective of this study was to investigate associations rather than estimate population parameters, this characteristic is unlikely to substantially affect the internal validity of the observed relationships [57,58].

A potential limitation of the present study is the assessment of habitual dietary intake using the KomPAN questionnaire, which evaluates only the frequency of food consumption and does not account for portion sizes or nutrient intake. As principal component analysis identifies dietary patterns based on the shared variance among variables, food groups characterised by low variability in consumption or weak intercorrelations may fail to achieve substantial factor loadings, and the resulting patterns are influenced by the type and quantification of the input variables [59,60,61]. This methodological characteristic may partly explain why 14 of the 33 analysed food groups were not retained within any of the identified dietary patterns and should therefore be taken into consideration when interpreting the findings. Nevertheless, the use of a validated frequency-based dietary questionnaire enabled the assessment of a broad spectrum of habitual dietary behaviours and remains an appropriate tool for exploratory dietary pattern analyses in population-based studies [59,60].

The absence of associations between dietary patterns, quality of life, and fibromyalgia symptoms may be explained by the predominant influence of psychosocial and functional factors. In the regression analysis, functional limitations, as well as psychological and social functioning, were the strongest determinants of quality of life, whereas dietary patterns did not significantly increase the model’s explained variance. These findings are consistent with studies indicating that the well-being of individuals with fibromyalgia is more strongly related to chronic functional impairment and psychological status than to diet alone [4,5]. The interpretation of these findings should also consider that dietary assessment was based on habitual food consumption patterns rather than on validated dietary inflammatory indices or objective biomarkers of systemic inflammation. Consequently, the study was designed to evaluate behavioural dietary patterns instead of the biological effects of diet, which limits conclusions regarding potential inflammatory mechanisms linking nutrition with quality of life in fibromyalgia. Future studies combining dietary pattern analysis with dietary inflammatory indices and inflammatory biomarkers, such as C-reactive protein, interleukin-6, or tumour necrosis factor-α, may provide a more comprehensive understanding of the relationship between nutrition, inflammation, and patient-reported outcomes [10,11,12].

## 5. Conclusions

The present study indicates that the quality of life of women with fibromyalgia is more closely associated with psychosocial functioning and disease-related functional limitations than with dietary patterns or anthropometric characteristics. Although self-rated economic status was associated with quality of life in the initial analyses, its importance was attenuated after accounting for clinical and psychosocial factors, suggesting that psychosocial functioning and the impact of the disease on daily activities are more strongly related to perceived quality of life.

No independent associations were identified between dietary patterns and self-rated quality of life. However, this finding should not be interpreted as evidence against dietary management. Healthy dietary habits, including dietary patterns with anti-inflammatory characteristics, should be regarded as complementary components of comprehensive lifestyle management that support overall health and symptom control. Therefore, multidisciplinary care integrating psychological support, social interventions, functional rehabilitation, and nutritional counselling may provide the most appropriate biopsychosocial approach for women with fibromyalgia.

## Figures and Tables

**Table 1:** Factor loading matrix for dietary patterns (factors) identified using principal component analysis (PCA).

Food Groups	Factors/Dietary Patterns			
	1	2	3	4
White bread, e.g., wheat, rye, mixed wheat–rye bread, toast bread, bread rolls, croissants	0.642	0.225	0.086	−0.075
Oils, margarines, or butter–margarine blends used as spreads or for frying, baking, and cooking purpose	0.630	−0.007	−0.044	−0.019
Fried dishes (meat-based and flour-based)	0.606	0.353	0.014	0.162
White rice, regular pasta, or fine groats, e.g., semolina, couscous, Kraków-style groats	0.596	−0.055	0.213	0.103
Potatoes (excluding chips and crisps)	0.526	0.214	0.061	0.059
Sweets, e.g., candies, biscuits, cakes, chocolate bars, and other confectionery products	0.442	0.085	−0.003	0.263
Sweetened hot beverages such as tea, coffee, herbal infusions, or fruit infusions	0.409	0.183	−0.204	0.088
Processed meat products, including sausages and frankfurters	0.126	0.727	−0.014	0.069
Dishes prepared from so-called red meat, e.g., pork, beef, veal, mutton, lamb, and game meat	0.084	0.633	−0.196	0.059
Yellow cheeses (including processed and blue cheeses)	0.152	0.583	0.220	0.117
Butter used as a spread or for frying, baking, and cooking purposes	0.286	0.471	0.018	0.333
Fruit	0.117	0.144	0.697	0.021
Vegetables	−0.072	−0.006	0.680	0.021
Wholegrain bread	0.090	−0.022	0.625	0.224
Water, e.g., mineral or table water	−0.082	0.042	0.484	0.047
Buckwheat groats, oatmeal, wholegrain pasta, or other coarse groats	0.069	−0.342	0.471	0.352
Cottage cheeses (including homogenised, curd, and cottage cheese desserts)	−0.092	0.161	0.049	0.791
Fermented dairy beverages, e.g., yoghurts and kefirs (plain or flavoured)	−0.005	0.101	0.180	0.778
Milk (including flavoured milk, cocoa, and coffee with milk)	0.333	0.074	0.076	0.598
% of explained variance	13.1	8.7	7.2	5.4

**Table 2:** Sociodemographic characteristics of the study group.

Characteristics		% (N) *
Total		100.0 (201)
Age	35 years and below	18.9 (38)
	36–45 years	29.4 (59)
	46–55 years	33.3 (67)
	Above 55 years	18.4 (37)
Education	Primary	1.0 (2)
	Vocational	6.0 (12)
	Secondary	32.3 (65)
	Higher	60.7 (122)
Place of residence	Rural area	27.4 (55)
	A town with fewer than 100,000 inhabitants	24.8 (50)
	City with more than 100,000 inhabitants	47.8 (99)
Family status	I live alone	13.4 (27)
	I live with my partner	39.8 (79)
	I live with my family, without my partner	12.4 (25)
	I live with my partner and my family	34.8 (70)
Self-reported financial situation	I live in poverty	2.5 (5)
	I live very modestly	15.4 (31)
	I live moderately	48.2 (97)
	I live well	29.9 (60)
	I live very well	4.0 (8)
Employment Status	I am retired or receiving a disability pension	14.4 (29)
	I am unemployed	26.4 (53)
	I am employed on a temporary or occasional basis and do not receive a pension or disability benefit	7.0 (14)
	I am employed on a temporary or occasional basis but receive a pension or disability benefit	2.0 (4)
	I have permanent employment	50.2 (101)
Financial support from family	There is no such need, as I am satisfied with my situation	38.8 (78)
	No, despite experiencing financial difficulties	14.4 (29)
	Yes, due to financial difficulties	34.9 (70)
	Yes, despite not experiencing financial difficulties	11.9 (24)
Use of social welfare services due to financial difficulties	There is no such need, as I am satisfied with my situation	58.7 (119)
	No, despite experiencing financial difficulties	34.3 (69)
	Yes, due to financial difficulties	6.5 (13)
	Yes, despite not experiencing financial difficulties	0.5 (1)
Use of social welfare services due to health-related problems	There is no such need, as my health allows me to function properly within the community	26.4 (53)
	No, despite experiencing health-related problems	65.2 (131)
	Yes, due to health-related problems	8.5 (17)
	Yes, despite not experiencing health-related problems	0.0 (0)

* N—number of respondents.

**Table 3:** Statistics on the variables on quality of life and fibromyalgia.

		Perceived Quality of Life		Quality of Life in Psychological Domain		Quality of Life in Social Domain		FIQR-Functioning		FIQR-Overall Impact		FIQR-Symptoms		FAS	
		Statistics	St. Error	Statistics	St. Error	Statistics	St. Error	Statistics	St. Error	Statistics	St. Error	Statistics	St. Error	Statistics	St. Error
Mean		2.42	0.059	10.52	0.129	10.61	0.218	20.08	0.383	14.89	0.280	35.37	0.471	25.02	0.430
95% confidence interval for the mean	Lower limit	2.31		10.26		10.18		19.32		14.34		34.44		24.18	
	Upper limit	2.54		10.77		11.06		20.83		15.44		36.30		25.87	
Median		2.00		11.00		11.00		21.00		16.00		36.25		25.00	
Variance		0.73		3.47		9.85		30.45		16.28		46.20		36.16	
Std. dev.		0.85		1.86		3.14		5.52		4.03		6.80		6.01	
Minimum		1.00		5.00		4.00		5.33		3.00		15.50		10.00	
Maximum		4.00		15.00		20.00		28.67		20.00		49.00		39.00	
Range		3.00		10.00		16.00		23.33		17.00		33.50		29.00	
Interquartile range		1.00		3.00		5.00		7.58		5.75		9.38		8.00	
Skewness		0.24	0.169	−0.19	0.169	0.12	0.169	−0.69	0.169	−0.82	0.169	−0.41	0.169	−0.16	0.169
Kurtosis		−0.54	0.336	−0.04	0.336	−0.23	0.336	−0.20	0.336	−0.01	0.336	0.08	0.336	−0.46	0.336

**Table 4:** Association between self-rated overall quality of life and selected variables—hierarchical regression models.

	B (SE)	β (Beta)	*p*-Value
Model 1—F(3:198) = 8.357; *p* < 0.001; R^2^_adj._ = 0.099			
Constant	1.708 (0.389)		<0.001
Age	−0.001 (0.005)	−0.018	0.799
Self-reported financial situation	0.333 (0.069)	0.325	<0.001
BMI	−0.010 (0.010)	−0.069	0.329
Model 2—F(7:194) = 3.896; *p* < 0.001; R^2^_adj._ = 0.092			
Constant	1.745 (0.414)		<0.001
Age	0.000 (0.006)	−0.004	0.953
Self-reported financial situation	0.319 (0.072)	0.311	<0.001
BMI	−0.011 (0.010)	−0.078	0.274
Factor 1	−0.015 (0.060)	−0.017	0.808
Factor 2	0.005 (0.057)	0.006	0.925
Factor 3	0.087 (0.058)	0.102	0.139
Factor 4	−0.020 (0.059)	−0.023	0.737
Model 3—F(11:190) = 9.432; *p* < 0.001; R^2^_adj._ = 0.317			
Constant	4.209 (0.506)		<0.001
Age	0.003(0.005)	0.037	0.582
Self-reported financial situation	0.108 (0.069)	0.105	0.118
BMI	0.001 (0.009)	0.009	0.887
Factor 1	−0.045 (0.052)	−0.053	0.392
Factor 2	−0.003 (0.050)	−0.003	0.956
Factor 3	0.042 (0.051)	0.049	0.419
Factor 4	−0.048 (0.051)	−0.056	0.353
FIQR Functioning	−0.031 (0.014)	−0.202	0.030
FIQR Overall Impact	−0.047 (0.019)	−0.220	0.015
FIQR Symptoms	−0.018 (0.011)	−0.146	0.083
FAS	−0.012 (0.009)	−0.089	0.192
Model 4—F(13:188) = 11.401; *p* < 0.001; R^2^_adj._ = 0.403			
Constant	2.347 (0.626)		<0.001
Age	0.001 (0.005)	0.013	0.838
Self-reported financial situation	0.060 (0.065)	0.059	0.355
BMI	0.000 (0.009)	0.003	0.958
Factor 1	−0.044 (0.049)	−0.052	0.373
Factor 2	0.009 (0.047)	0.011	0.845
Factor 3	0.032 (0.048)	0.038	0.512
Factor 4	−0.046 (0.048)	−0.054	0.342
FIQR Functioning	−0.031 (0.013)	−0.202	0.022
FIQR Overall Impact	−0.040 (0.018)	−0.189	0.026
FIQR Symptoms	−0.002 (0.011)	−0.014	0.866
FAS	−0.013 (0.009)	−0.095	0.134
Quality of life in the social domain	0.049 (0.021)	0.162	0.023
Quality of life in the psychological domain	0.086 (0.024)	0.234	<0.001

F—ANOVA test statistic value; R^2^_adj._—adjusted R-squared value; B—unstandardised regression coefficient; SE—standard error; β (Beta)—standardised regression coefficient.

**Table 5:** Summary of the Regression Models.

Model	Variables	R^2^	R^2^_adj._	R^2^ Change
1	Age, BMI, Self-reported financial situation	0.113	0.099	
2	Variables from model 1, Dietary patterns	0.124	0.092	0.012
3	Variables from model 2, FAS, FIQR Overall Impact, FIQR Symptoms, FIQR Functioning	0.354	0.317	0.230
4	Variables from Model 3, Quality of life in the social domain, Quality of life in the psychological domain	0.442	0.403	0.088

## Data Availability

The research data are available in the Knowledge Base Repository of the Wrocław University of Environmental and Life Sciences at: https://bazawiedzy.upwr.edu.pl/info/researchdata/UPWRcbe0b3d7e06e4e5cbf292fc7700fce80/ (accessed on 20 May 2026); DOI: https://doi.org/10.57755/48gw-d684.
